# How do El Niño Southern Oscillation (ENSO) and local meteorological factors affect the incidence of seasonal influenza in New York state

**DOI:** 10.1016/j.heha.2022.100040

**Published:** 2022-11-28

**Authors:** Jianpeng Xiao, Michael Gao, Miaoling Huang, Wangjian Zhang, Zhicheng Du, Tao Liu, Xiaojing Meng, Wenjun Ma, Shao Lin

**Affiliations:** a Guangdong Provincial Institute of Public Health, Guangdong Provincial Center for Disease Control and Prevention, Guangzhou 511430, China; b Department of Occupational Health and Occupational Medicine, Guangdong Provincial Key Laboratory of Tropical Disease Research, School of Public Health, Southern Medical University, Guangzhou 510515, China; c Department of Environmental Health Sciences, School of Public Health, University at Albany, State University of New York, Rensselaer, NY 12144, United States; d Department of Obstetrics and Gynecology, Sun Yat-sen Memorial Hospital, Sun Yat-sen University, Guangzhou 510120, China; e Department of Medical Statistics and Epidemiology, School of Public Health, Sun Yat-sen University, Guangzhou 510080, China; f Department of Public Health and Preventive Medicine, School of Medicine, Jinan University, Guangzhou 510632, Guangdong, China

**Keywords:** El Niño southern oscillation, Weather, Influenza, Wavelet analysis, Association

## Abstract

**Background:**

Research is lacking in examining how multiple climate factors affect the incidence of seasonal influenza. We investigated the associations between El Niño Southern Oscillation (ENSO), meteorological factors, and influenza incidence in New York State, United States.

**Method:**

We collected emergency department visit data for influenza from the New York State Department of Health. ENSO index was obtained from the National Oceanic and Atmospheric Administration. Meteorological factors, Google Flu Search Index (GFI), and Influenza-like illness (ILI) data in New York State were also collected. Wavelet analysis was used to quantitatively estimate the coherence and phase difference of ENSO, temperature, precipitation, relative humidity, and absolute humidity with emergency department visits of influenza in New York State. Generalized additive models (GAM) were employed to examine the exposure-response relationships between ENSO, weather, and influenza. GFI and ILI data were used to simulate synchronous influenza visits.

**Results:**

The influenza epidemic in New York State had multiple periodic and was primarily on the 1-year scale. The incidence of influenza closely followed the low ENSO index by an average of two months, and the lag period of ENSO on influenza was shorter during 2015–2018. Low temperature in the previous 2 weeks and low absolute humidity in the prior week were positively associated with influenza incidence in New York State. We found an l-shaped association between ENSO index and influenza, a parabolic relationship between temperature in the previous two weeks and influenza, and a linear negative association between absolute humidity in the previous week and influenza. The simulation models including GFI and ILI had higher accuracy for influenza visit estimation.

**Conclusions:**

Low ENSO index, low temperature, and low absolute humidity may drive the influenza epidemics in New York State. The findings can help us deepen the understanding of the climate-influenza association, and help to develop an influenza forecasting model.

## 1. Introduction

The disease burden and economic costs of the influenza epidemic have become a major challenge for both clinical settings and public health. Seasonal influenza (flu) is a viral respiratory illness with reservoirs in avian and mammalian species. Globally, seasonal influenza epidemics are estimated to result in up to 650,000 deaths per year (Lee et al., 2018). The Centers for Disease Control and Prevention (CDC) reported that influenza had caused 12,000–79,000 deaths annually in the United States (US) since 2010 (USCDC, 2018). The number of cases in the 2017–2018 influenza season was about 49.0 million, the highest since the 2009 H1N1 pandemic during which about 60.8 million people in the US were infected with influenza (Shrestha et al., 2011).

The high incidence of the 2017–2018 influenza epidemics in the US underscored that influenza epidemics vary by year. However, the determinants of such time-varying in the influenza epidemic are unknown. One potential determinant is El Niño Southern Oscillation (ENSO), a time-varying climatic phenomenon that produces inter-annual changes in temperature and precipitation (Ebi et al., 2001; Oluwole, 2015). Shaman and Lipsitch report that the four most recent human influenza pandemics were preceded by the cold phase of ENSO (La Niña), and that low ENSO index may be associated with influenza pandemics (Shaman and Lipsitch, 2013). Viboud et al. reported that the magnitude of ENSO is associated with morbidity in influenza epidemics and that ENSO may be a driver of the epidemic (Viboud et al., 2004). Whereas, Fisman et al. did not find that ENSO had an impact on the incidence of pneumonia and influenza in the US (Fisman et al., 2016). Generally, the relationship between ENSO and influenza is under-studied, and the previous findings are controversial. Additionally, the potential lag effect of ENSO on influenza is little understood.

ENSO has pronounced global and regional circulation effects, which may lead to regional climate fluctuations (Patz et al., 2005;Dogar et al., 2017). During the regional climate fluctuations, local weather conditions may impact the incidence of seasonal influenza (Davis et al., 2012). Studies have reported associations between influenza and meteorological factors. For instance, decreases in temperature and absolute humidity (AH) are associated with increases in risk for influenza (Jaakkola et al., 2014). Moreover, high absolute humidity was found to inhibit both influenza transmission and virus survival more significantly than relative humidity (RH) (Jeffrey and Melvin, 2009;Peci et al., 2019). However, the exposure-response relationships between multiple meteorological factors such as absolute humidity and influenza incidence are less examined. In addition, several studies found that the Google Flu trend was correlated with the influenza-like activity level (Dugas et al., 2012;Olson et al., 2013;Araz et al., 2014), while the association was inconsistent (Klembczyk et al., 2016;Kandula and Shaman, 2019). Understanding these relationships would aid the development of influenza forecasts.

To fill the knowledge gaps described above, we aim to investigate the lag correlation and exposure-response associations between ENSO, multiple meteorological factors, and the influenza incidences in New York State (NYS) which is an important epidemic region of influenza in the US. We also assessed the accuracies of using Google Flu Search Index (GFI) and Influenza-like illness (ILI) data to predict influenza incidences.

## 2. Methods

### 2.1. Data sources

Climate and weather datasets: (1) ENSO index data from January 2005 through June 2018 was collected from the National Oceanic and Atmospheric Administration (NOAA) (https://origin.cpc.ncep.noaa.gov). ENSO index is an indicator of ENSO events that are based on sea surface temperature (SST) in the east-central tropical Pacific Ocean. (2) Meteorological factors including temperature, precipitation and relative humidity (RH) in NYS were collected from the NOAA (https://www.noaa.gov/weather). (3) Since absolute humidity data was not directly available from NOAA, we calculated it through the method of a previous report based on daily temperature and relative humidity (Carnotcycle., 2012).

Influenza datasets: (1) Emergency department (ED) visit data for influenza in NYS from 1/1/2005 through 6/30/2017 were obtained from the Statewide Planning and Research Cooperative System (SPARCS) operated by the NYS Department of Health (https://www.health.ny.gov/statistics/sparcs). SPARCS collects information on over 95% of ED visits in NYS, including the principal and up to 24 other diagnoses, date of birth, street address, and admission date (Xiao et al., 2021). The data of ED visits for influenza from July 2017 to June 2018 in NYS was not available during our study. Influenza-like illness (ILI) and Google flu search index (GFI) have been found useful signals of influenza epidemics (Araz et al., 2014;Zhang et al., 2018;USCDC, 2019). Hence, we used Influenza-like illness and Google flu search index data to simulate ED visits for that period. (2) Influenza-like illness data in NYS was collected from the US Outpatient Influenza-like Illness Surveillance Network (ILINet) (https://www.cdc.gov/flu). (3) Google flu search index in NYS was obtained from Google Trends (https://trends.google.com).

#### 2.2.1. Exposure definitions

The ENSO index used in this study is the sea surface temperature (SST) anomaly index (also named Oceanic Niño Index (ONI)) for Niño region 3.4 (5°N to 5°S, 170°W to 120°W), which was a 3-month moving average based on centered 30-year base periods (https://origin.cpc.ncep.noaa.gov/products/analysis_monitoring/ensostuff/ONI_v5.php). El Niño (La Niña) is a phenomenon in the equatorial Pacific Ocean characterized by a five consecutive SST is above (below) the threshold of +0.5 °C (−0.5 °C) (https://www.ncdc.noaa.gov/teleconnections/enso/sst). To match the dataset, the daily average temperature, cumulative precipitation, average RH and average AH in NYS at the state-level were aggregated by monthly data when conducting a wavelet analysis and were aggregated by weekly data when conducting a generalized additive model analysis.

#### 2.2.2. Outcome definitions

Influenza was diagnosed by a clinician based on the clinical manifestations or laboratory test of an emergency department (ED) visitor. Influenza included in this study was defined as a primary diagnosis reported as International Classification of Diseases, Ninth Revision, Clinical Modification (ICD-9-CM) Diagnosis Code 487.0 or ICD-10-CM Diagnosis Code J09. Google flu search index was defined as the number of Google searches for “influenza” in NYS. The Influenza-like illness data used in this study was the percentage of patient visits to healthcare providers in NYS for Influenza-like illnesses reported from the ILINet. Google flu search index and Influenza-like illness were recorded by week and were aggregated to monthly data when conducting a wavelet analysis.

### 2.3. Statistical analyses

We employed wavelet analysis to explore the coherence and time-lag between time series of exposures (ENSO and weather) and the outcome (influenza). Then we investigated the exposure-response association between he ENSO index, meteorological factors, and influenza incidences using generalized additive models (GAM). The research framework in this study was shown in Figure S1.

#### 2.3.1. Wavelet analysis

Wavelet analysis involves the transformation of a data series with a localized wave for decomposition and reconstruction across multiple timescales, which makes it a powerful tool for time-frequency analysis of non-stationary data. We use wavelet analysis to investigate the coherence and time-lag phases for ENSO-influenza and weather-influenza in NYS.

All time-series were square-root transformed and normalized before wavelet transformation to reduce skewing, in line with Cazelles’s reports (Cazelles et al., 2005). In the initial study, we observed that low ENSO index, low temperature or low humidity seem to lead influenza incidence in NYS. Thus, ENSO index, temperature and humidity were converted to inverse, to facilitate displaying the wavelet coherency between them and influenza. The Morlet Wavelet Transform was then used to identify time-frequencies for each analyzed time series. The wavelet power spectrum for each time series was displayed in image plots for the periodicity of the fitted series according to the previous study (Xiao et al., 2018). Next, cross-wavelet transform was used to compare pairs of time series, to identify co-variability and the phase association. Finally, wavelet coherency was used to identify significant cross-correlation between these time series and phase differences. We computed the difference in phase angles (degrees) and phase period (days) between time series, and produced a comparative plot of phase paths for selected periods.

To explore the potential pathway from ENSO index to influenza incidence, we also conducted a cross-correlation analysis among ENSO index, temperature, absolute humidity, and influenza incidence at lag 1–4 months, and performed a wavelet coherency analysis of ENSO with temperature and absolute humidity, respectively

#### 2.2.2. Generalized additive model (GAM) analysis

The above wavelet analysis implied that low ENSO index, low temperature, and low absolute humidity may lead to influenza incidence. To further investigate the exposure-response relationship between ENSO and influenza, weather and influenza, we conducted a Generalized additive model (GAM) analysis based on weekly data. We used a cubic spline function of variables and applied a quasi-Poisson model to allow for over-dispersion of the data. The model was specified as follows:

$$Log\left(u_{t}\right)=\beta_{0}+s\left(\text{Variabl}e_{t-e},df\right)+s\left(\text{time},df\right)+offset\left(POP\right)$$

Where *u*_*t*_ is expected influenza visits on week *t*, *s*(*Variables*_*t* −*e*,_ *df*) denotes the cubic spline of ENSO index, temperature, or absolute humidity in the previous *e* weeks with the corresponding *df of 3*, and where *s (time, df)* is the cubic spline smoothing function of the time with *df* of 1 per year to control seasonal and long-term trends. *offset (POP)* is an offset of population by year.

#### 2.3.3. Bayesian structural time series (BSTS) model analysis

Bayesian structural time series models are ideal for short-term prediction or simulation (Scott and Varian, 2013;Brodersen et al., 2015). To complement the existing influenza visit data, we used BSTS models to simulate influenza visit data for the inaccessible period of July 2017 to June 2018 in New York State.

The model was trained with monthly influenza visit data and two covariates, Influenza-like illness and Google flu search index spanning October 2010 to June 2017. There were three models in this study: a model adjusted to Influenza-like illness, a model adjusted to the Google Flu index, and a model adjusted to both Influenza-like illness and Google flu index. We first used a dataset spanning October 2010 to June 2016 to train the models and for validation, and then simulated the ED visits of influenza from July 2016 to June 2017. Root mean square error (RMSE) was used to assess the fitness of the models, and we selected the model with the lowest value of RMSE from the three models. Finally, this model was used to simulate the ED visits from July 2017 to June 2018 in New York State.

In the present study, Wavelet analyses, GAM analysis, and BSTS analysis were performed using the “*WaveletComp*”, “*mgcv*”, and “*Counterfactual*” packages in R soft 3.6.1 (Core, 2015), respectively.

## 3. Results

### 3.1. Basic information

There were 122,477 emergency department visits for influenza in New York State from January 2005 to June 2017. Influenza transmission followed a seasonal pattern where the peak epidemic period was typically from December to February of the next year. However, the 2009 epidemic did not entirely follow this pattern. Temperature, precipitation and humidity also showed seasonal variation (Figure S2). Influenza-like illness and Google flu index highly corresponded to the visits of influenza. The ENSO index decreased markedly in the winters of 2007, 2008, 2010, 2011 and 2017.

### 3.2. Periodicity of influenza epidemics

**Fig. 1.**
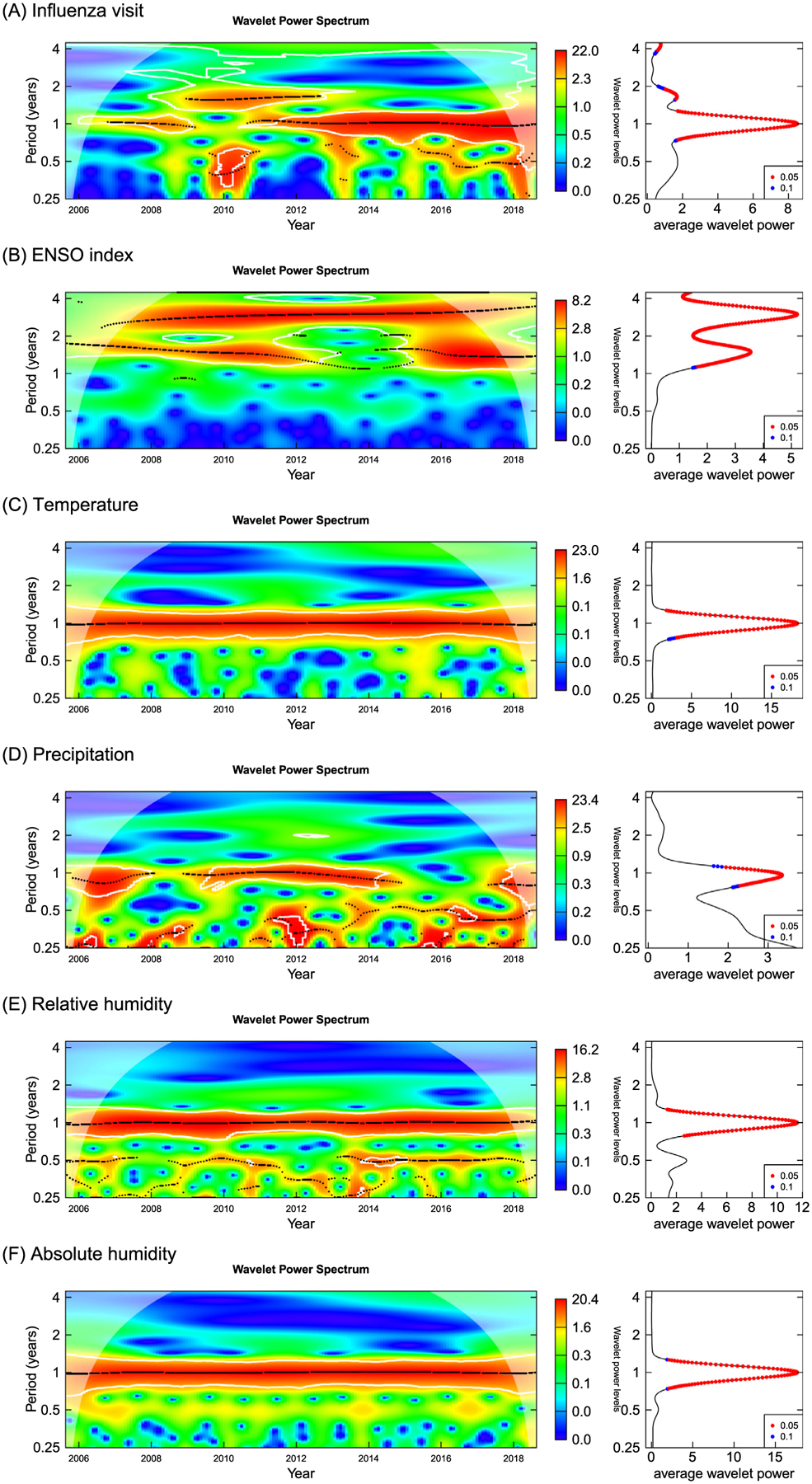
Wavelet spectra of monthly influenza incidence, ENSO index, mean temperature, precipitation, mean relative humidity, mean absolute humidity and ENSO index during 2005–2018. (A) Influenza visit; (B) ENSO index; (C) Temperature; (D) Precipitation; (E) Relative humidity; (F) Absolute humidity.

Fig. 1 shows the wavelet power spectrum for monthly influenza incidence, ENSO index, temperature, precipitation, relative humidity, absolute humidity during 2005–2018. The wavelet transforms of influenza incidence, precipitation, relative humidity, and absolute humidity displayed significant periodicity on the 1-year scale. 1.5–2 years period and 2.5–3.0 years period were observed for the ENSO index respectively for 2006–2012 and 2008–2017, but the average power was poor (Fig. 1 B).

### 3.3. Cross-correlation and phase differences

**Fig. 2.**
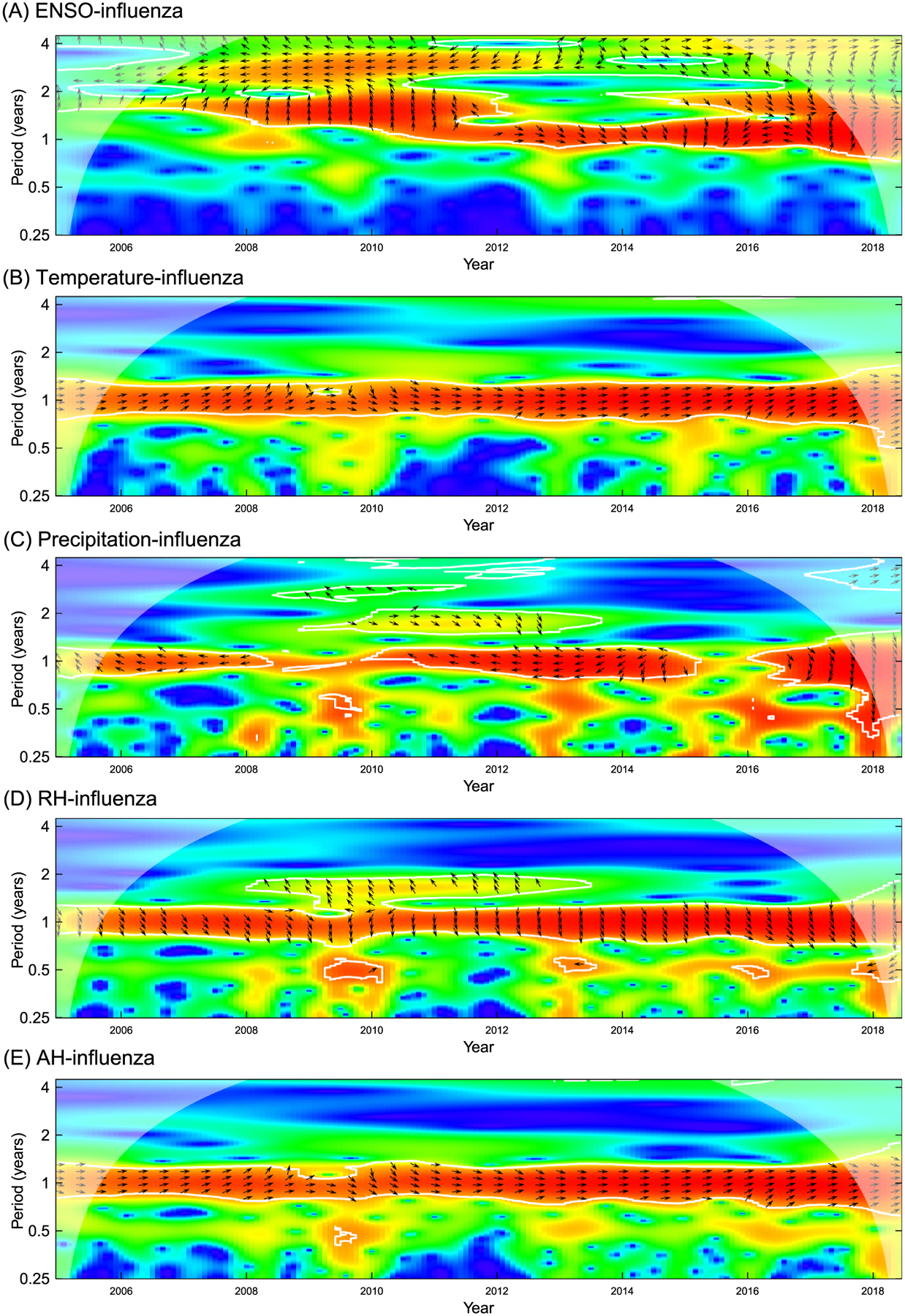
The cross wavelet spectra for ENSO-, temperature-, precipitation-, relative humidity (AH)-, and absolute humidity (AH)-influenza. (A) ENSO-influenza. (B) temperature-influenza. (C) precipitation-influenza. (D) RH-influenza. (E) AH-influenza.

The cross wavelet power spectra for temperature-, precipitation-, relative humidity-, and absolute humidity-influenza showed that regions in the time-frequency space displayed high common power in the 1-year scales (Fig. 2B, C, D, E) while the spectra for ENSO-influenza had high power on the 1–4 year scale (Fig. 2A). The directions of the arrows for the spectra for temperature-influenza and absolute humidity absolute humidity-influenza generally point to the right and upward, indicating that ENSO index, temperature, and absolute humidity were in-phase with influenza incidence and that they may lead the influenza epidemics (Fig. 2A, B, E).

**Fig. 3.**
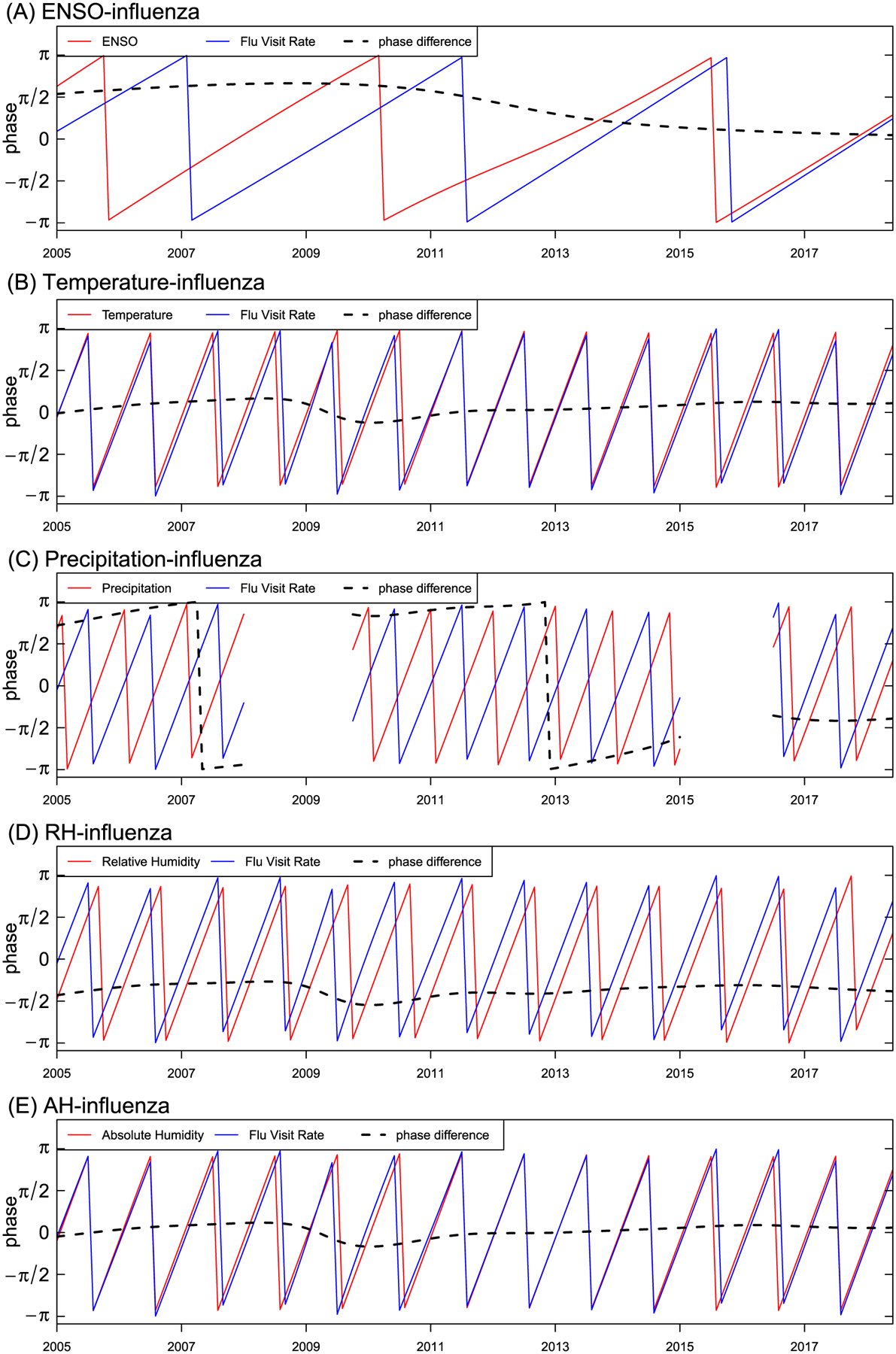
Phase differences between ENSO, temperature, precipitation, relative humidity (RH), absolute humidity (AH), and influenza incidence. (A) ENSO-influenza at a periodicity of 4-years. (B) temperature-influenza at a periodicity of 1-year. (C) precipitation-influenza at a periodicity of 1-year. (D) RH-influenza at a periodicity of 1-year. (E) AH-influenza at a periodicity of 1-year.

The phase differences between ENSO index, meteorological factors and influenza in 2005–2018 are shown in Fig. 3. We observed that low ENSO index, low temperature and low absolute humidity led the influenza incidences during the study period. Increased influenza incidence closely followed low ENSO index by approximately 2 months (65 days), low temperature by about 2 weeks (15 days), and low absolute humidity by about 1 week (8 days) (Table 1). Whereas, low relative humidity lagged behind influenza incidence by 2 months.

For the relationship between ENSO events and local weather, we found that both temperature and humidity were generally higher during/following the El Niño events and lower during/following the La Niño events compared with reference periods (Figure S3), suggesting potential associations between ENSO events and weather. According to the result of cross-correction analysis and wavelet analysis, we found that the local temperature or absolute humidity has a moderate correlation with the ENSO index, and the impact of ENSO index on temperature or absolute humidity in New York State is delayed by an average of 1–2 months. (Figure S4, Figure S5).

### 3.4. Exposure-response relationship analysis

**Fig. 4.**
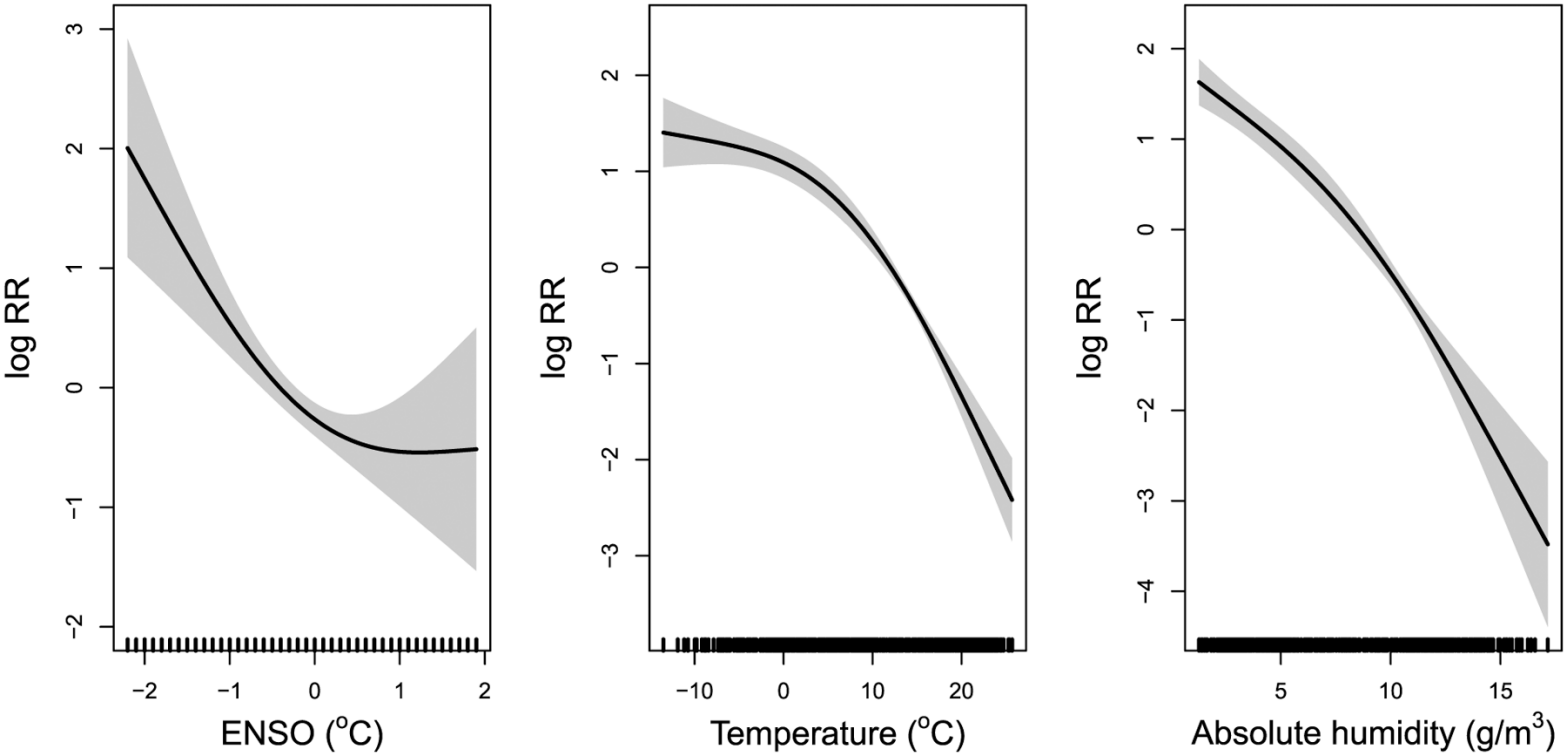
Exposure-response curves of ENSO index in the previous nine weeks, the temperature in the previous two weeks, and absolute humidity in the previous week against influenza incidence.

Fig. 4 shows the exposure-response curves of ENSO index, temperature, and absolute humidity against influenza incidence in New York State. An “L” shaped association was found between the ENSO index in the previous nine weeks and influenza incidence, with an inflection point of 0 °C for the ENSO index. An approximate parabolic relationship was found between temperature in the previous two weeks and influenza, with an inflection point near 0 °C. The risk for influenza decreases rapidly when the weekly temperature increases above 0 °C. An approximate negative linear relationship between absolute humidity in the previous week and influenza was observed.

### 3.5. Simulation for influenza visits

Table S1 shows the model fit for three BSTS models including Influenza-like illness, Google flu index, and both as predictors. The fitting of the model including Google flu index was better than that of the model including Influenza-like illness. When both Influenza-like illness and Google flu index were used as predictor variables, the fitting accuracy increased, reaching RMSE of 0.031 for the simulation period (Table S1 and Figure S6). Therefore, we simulated emergency department visits for influenza in New York State from July 2017 to June 2018 using the model with both Influenza-like illness and Google flu index. Figure S7 shows the trends in fitting and simulation from the three models. According to the simulation, about 52,729 visits occurred from July 2017 to June 2018, and the visit peaked in February 2018.

## 4. Discussion

### 4.1. Periodicity of influenza epidemics in New York state

The influenza incidence in New York State had multiple periodic modes of 0.5, 1, or 2 years, and was primarily on the 1-year scale. Our present analysis is in agreement with other previous studies that influenza has strong annual periodicity and weaker inter-seasonal periodicity (Tamerius et al., 2011). However, the inter-epidemic modes of influenza in New York State were shorter than those for countries in the northern hemisphere, which had periodicities of 12, 18, and 32 months (Oluwole, 2015). For instance, Japan, whose main islands are located in the northern hemisphere, has notably longer inter-epidemic modes of 3.1 and 4.0 years (Sumi et al., 2011). Multi-year periodicities exceeding 3.5 years were not entirely explicit in our 14-year data set, and our findings suggest that influenza incidence in New York State was not explicitly multi-year periodic during 2005–2018, further study with long-term data would reveal more multi-year periodicities for influenza in New York State.

### 4.2. ENSO and influenza

We found that the low ENSO index precedes influenza incidence in New York State, which implied that a low ENSO index might be associated with subsequent influenza. This result supports the finding that La Niña may drive the influenza epidemics (Shaman and Lipsitch, 2013), and is consistent with the finding that strong cold ENSO phases have been associated with larger and more severe influenza epidemics in France (Viboud et al., 2004). We also found a large decrease in lag time between low ENSO index and influenza incidence, which suggests that any impacts of ENSO on influenza are occurring faster. Furthermore, it has previously been proposed that La Niña may affect migratory patterns in reservoir species, and thereby increase the re-assortment of influenza virus (Shaman and Lipsitch, 2013). Regional changes in temperature, precipitation, and humidity may affect the capability of local ecological systems to support influenza reservoir species.

One mechanism by which low ENSO index may drive influenza in New York State is by causing weather conditions that enable influenza transmission or virus survival. Under this hypothesis, the impact of low ENSO index on influenza would be delayed by two months due to two steps across different periods: 1) the impact of low ENSO index on a local low temperature or low absolute humidity in New York State was delayed by average 1–2 months during the study period; and 2) influenza in New York State is highly correlated with the temperature in the prior 15 days and the absolute humidity in prior 5 days. This would explain that ENSO precedes influenza by an average of two months. Generally, our study implied a pathway of ENSO influencing the influenza incidence via local weather, and local weather would be the mediation factor for this impact. However, the physical mechanism underlying the multi-scale inter-actions between these components remains complex and unclear. More data from multiple regions would be needed for further clarification.

The exposure-response relationship between ENSO index and influenza highlights the potential role of low ENSO index in heightening influenza epidemics. In New York State, the risk increases exponentially as the ENSO index decreases below 0 °C. A study in California found that influenza mortality was significantly lower during El Niño, which supports our analysis that in aggregate, influenza incidence during El Niño (high ENSO index) is lower than during La Niña (low ENSO index) (Choi et al., 2006). Our findings add evidence that low ENSO index may drive influenza epidemic and ENSO index may be used as a predictor for influenza in the north of the US.

### 4.3. Meteorological factors and influenza

We noted that increased influenza visits followed low temperature by 15 days, which is in line with the finding by Davis et al. that influenza mortality lagged behind low temperature by 17 days in New York City (Davis et al., 2012). Gomez-Barroso et al. found that peaks in weekly influenza coincided with minimums for average temperature (Gomez-Barroso et al., 2017). Similarly, in New York State, increased influenza incidence followed decreases in absolute humidity by 8 days, akin to the findings of Shaman et al. that influenza epidemics tend to follow low absolute humidity conditions by two weeks in the US (Shaman et al., 2010). Differences in lag time between exposure to low absolute humidity to influenza virus infection, symptom onset, and emergency visits may account for this discrepancy (Lessler et al., 2009). We agree with previous studies that temperature and humidity may have separate impacts on influenza (Lowen and Steel, 2014). We observed that relative humidity did not lead influenza, and instead followed influenza by two months. Prior studies have found that absolute humidity explains a greater proportion of variation in influenza transmission efficiency and virus survival than relative humidity (Jeffrey and Melvin, 2009). We provide epidemiological evidence that absolute humidity, but not relative humidity, affect seasonal variations of influenza transmission.

In the current study, we found a nonlinear relationship between temperature and influenza, and temperature where 0 °C seems to be the turning point, which is similar to findings from other studies (Barreca and Shimshack, 2012;Peci et al., 2019). We also found absolute humidity has an approximately linear relationship with influenza, which is broadly in agreement with recent studies in the US (Tamerius et al., 2013;Peci et al., 2019). Tamerius et al. (Tamerius et al., 2013) estimated through logistic regression that the probability of virus survival and transmission decreased approximately linearly with increasing absolute humidity. This is supported by the work of Myatt et al. (Myatt et al., 2010), who found that an increase in absolute humidity was associated with a decrease in influenza virus survival. The findings indicate that low temperature and low absolute humidity would increase the risk of influenza transmission, and the temperature and absolute humidity may be useful factors for influenza forecasting.

### 4.4. Index for influenza epidemic

Our study found that a BSTS model incorporating both Google flu index and Influenza-like illness performed better than models using either Google flu index or Influenza-like illness alone. A study in Maryland found high correlations between Google flu trends and emergency department visits of influenza (Dugas et al., 2012). Similarly, Jeremy et al. reported that Google search queries might help detect influenza epidemics in areas with a large population of web search users (Ginsberg et al., 2009). Our results supported that Google search queries may be a good predictor for influenza incidence, especially when used in tandem with Influenza-like illnesses (Santillana et al., 2014). The BSTS model developed may be used to detect an influenza epidemic when the registered state data is not available yet or even to predict coming influenza epidemics.

### 4.5. Strengths and limitations

To the best of our knowledge, this is one of the few studies investigating the association between multiple climate factors, including ENSO index, meteorological factors and influenza incidence. Our study may also be the first study examining the lag effects of ENSO or other meteorological factors on influenza visits in New York State. On the other hand, this study has some potential limitations. First, this study used emergency department visits of influenza, which captures only the tip of the iceberg, covering only severe influenza cases resulting in emergency department or hospitalizations, and excluding less severe, non-ED influenza cases. Therefore, the total impact on all influenza cases may have been underestimated. However, the influenza visits in New York State exhibited high correlations with Google flu search index and Influenza-like illness, which have greater coverage of all cases. Second, the impact of ENSO may be masked by local variations in the type of influenza virus in different years, vaccination rates, randomly concomitant epidemics, and other factors regulating influenza dynamics such as socio-ecological conditions that may require further studies. Third, the findings from New York State may not generalize to other regions with different climate or weather patterns. However, the study method we established can be applied to the rest of the United States, and our findings can be re-examined. Fourth, there were just a few ENSO events during the study period. A further study based on a longer multiyear ENSO life cycle would help to address the relationship between ENSO and influenza.

## 5. Conclusion

This study found that low ENSO index in the preceding two months may influence the influenza incidence in New York State. Low temperature and low absolute humidity were positively associated with influenza visits, with special exposure-response relationships. The findings can help us deepen the understanding of the climate-influenza association, help to develop an influenza forecasting model and provide implications for the related public-health decision.

## Supplementary Material

1

## Figures and Tables

**Table 1:** Phase differences between ENSO index, temperature, relative humidity (RH), absolute humidity (AH), and influenza in NYS.

Pairs	Angle difference	Mean difference (days)	Median difference (days)
ENSO – Influenza	71.5°	72	65
Temperature – Influenza	12.3°	12	15
RH – Influenza	−64.3°	−65	−61
AH – Influenza	5.2°	5	8
