# Supplementary material for: How do El Niño Southern Oscillation (ENSO) and local meteorological factors affect the incidence of seasonal influenza in New York state
Source: Hyg Environ Health Adv. Author manuscript; Available in PMC 2023 Feb 10. (PMC9914518; doi:10.1016/j.heha.2022.100040)
Supplement: 1 [file NIHMS1861415-supplement-1.docx]

**Supplemental Materials**

**How do El Niño Southern Oscillation (ENSO) and local meteorological factors affect the incidence of seasonal influenza**

**in New York State**

**
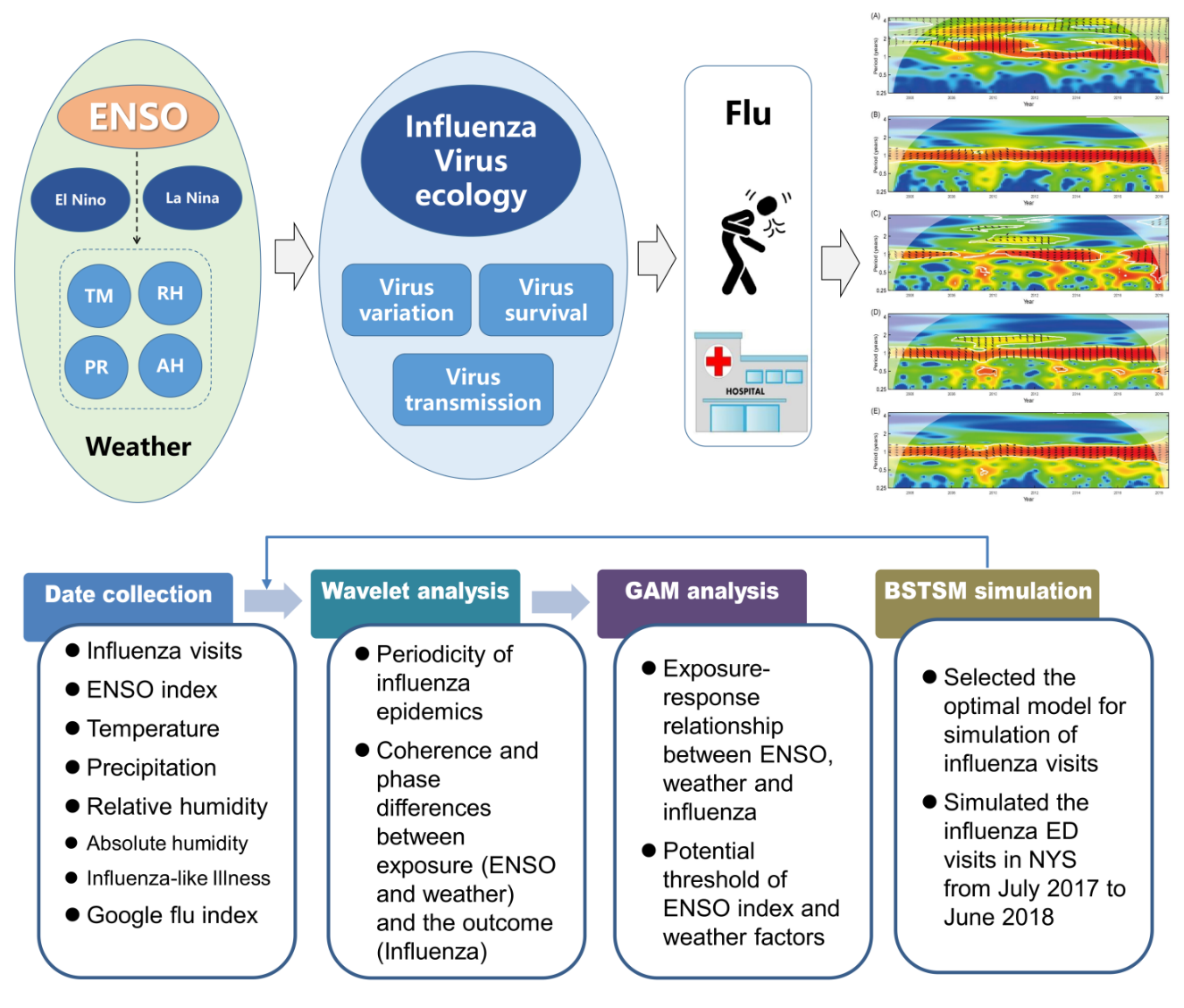
**

**Figure S1**. Research framework in this study

**
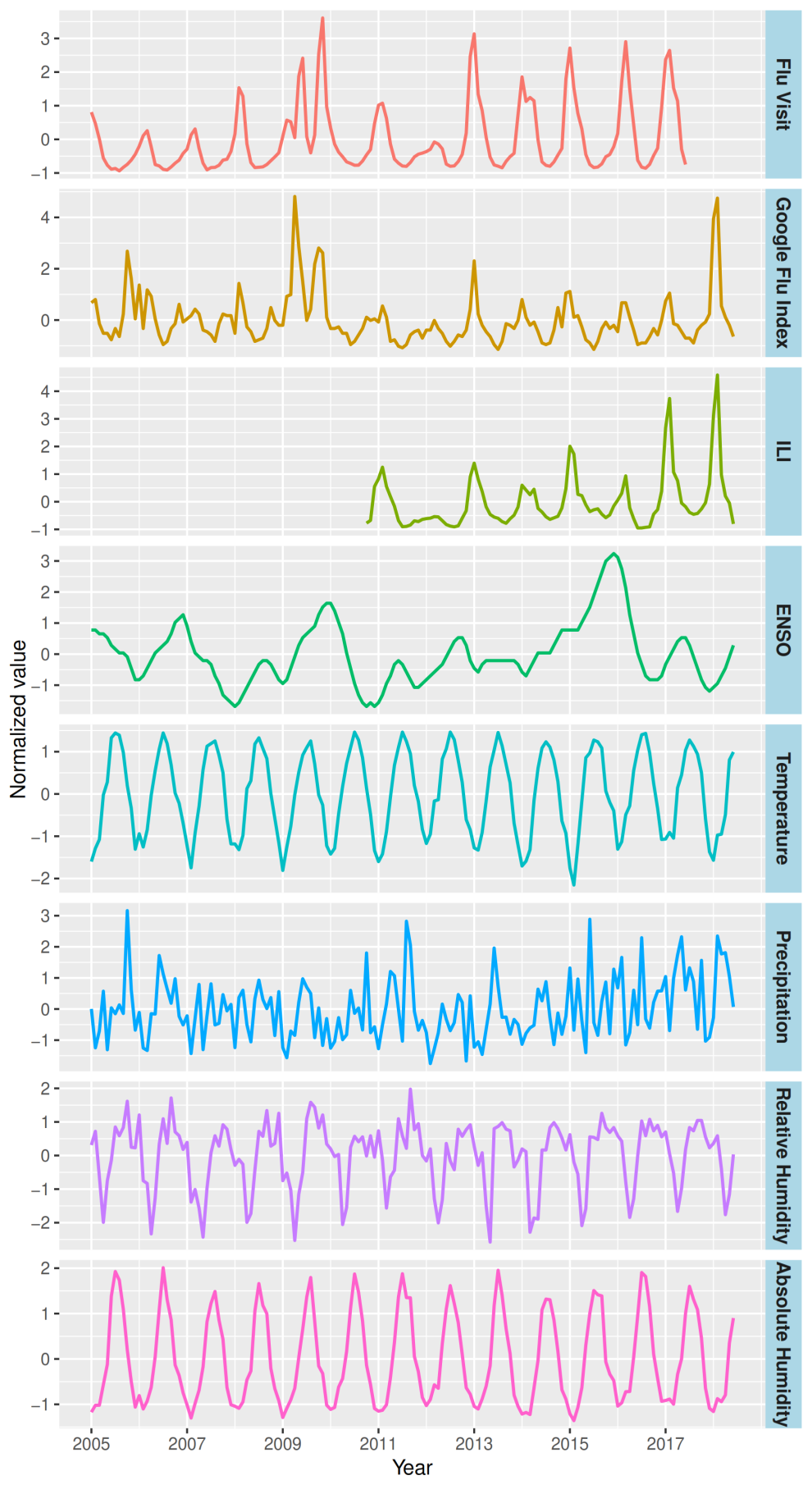
**

**Figure S2** Time-series of monthly influenza visits, Influenza-like illness, Google flu search index, ENSO index, temperature, precipitation, relative humidity, and absolute humidity in New York State in this study

**Figure S3** The time-series of ENSO index versus temperature, and ENSO index versus absolute humidity in New York State from January 2005 to June 2018 (The ENSO index used in this study is the sea surface temperature (SST) anomaly index for Niño region 3.4.)

**
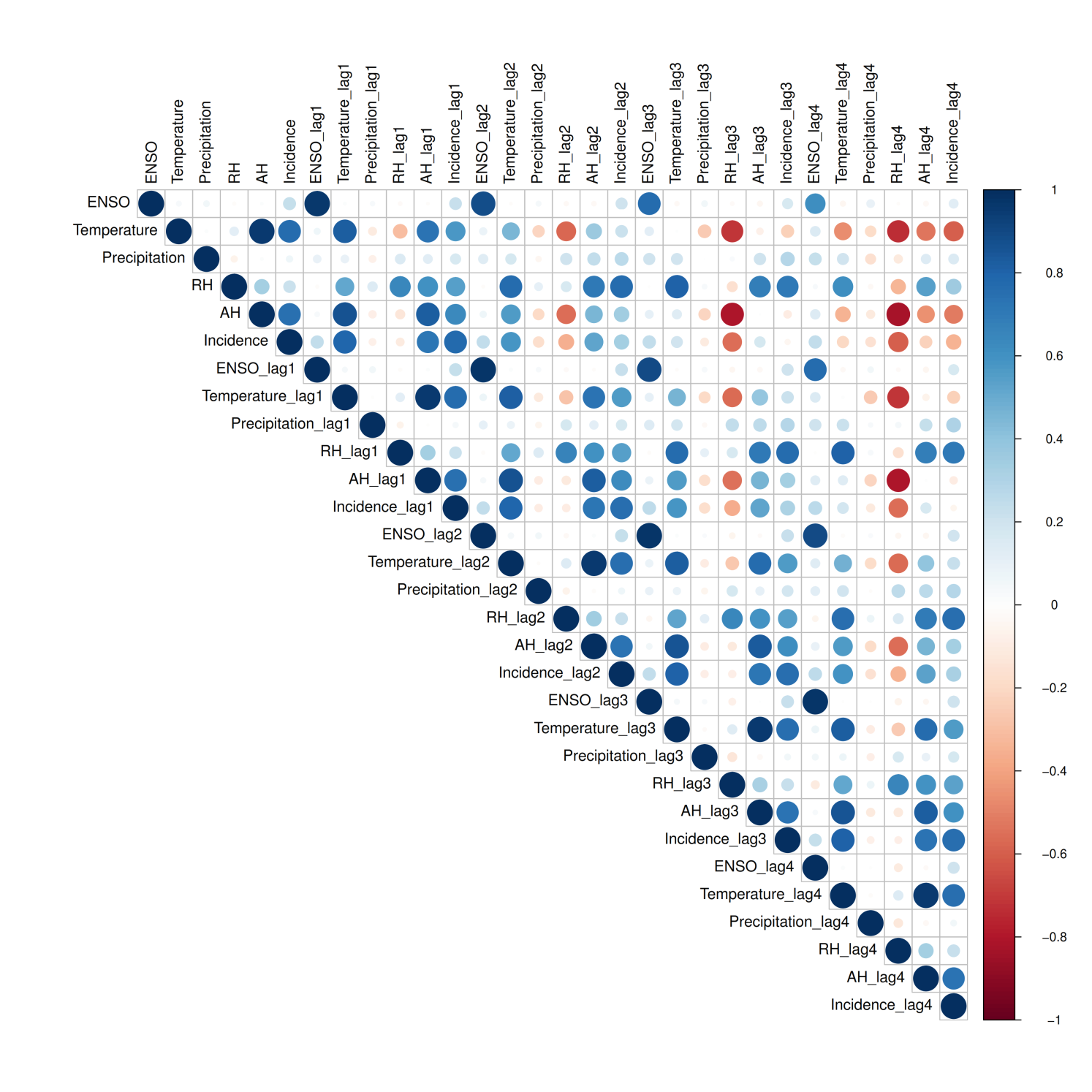
**

**Figure S4** The correction coefficient between ENSO index, meteorological factors, and influenza incidence at lag 1-4 months in New York State (ENSO index, temperature, relative humidity [RH], absolute humidity [AH] were converted to inverse)

**
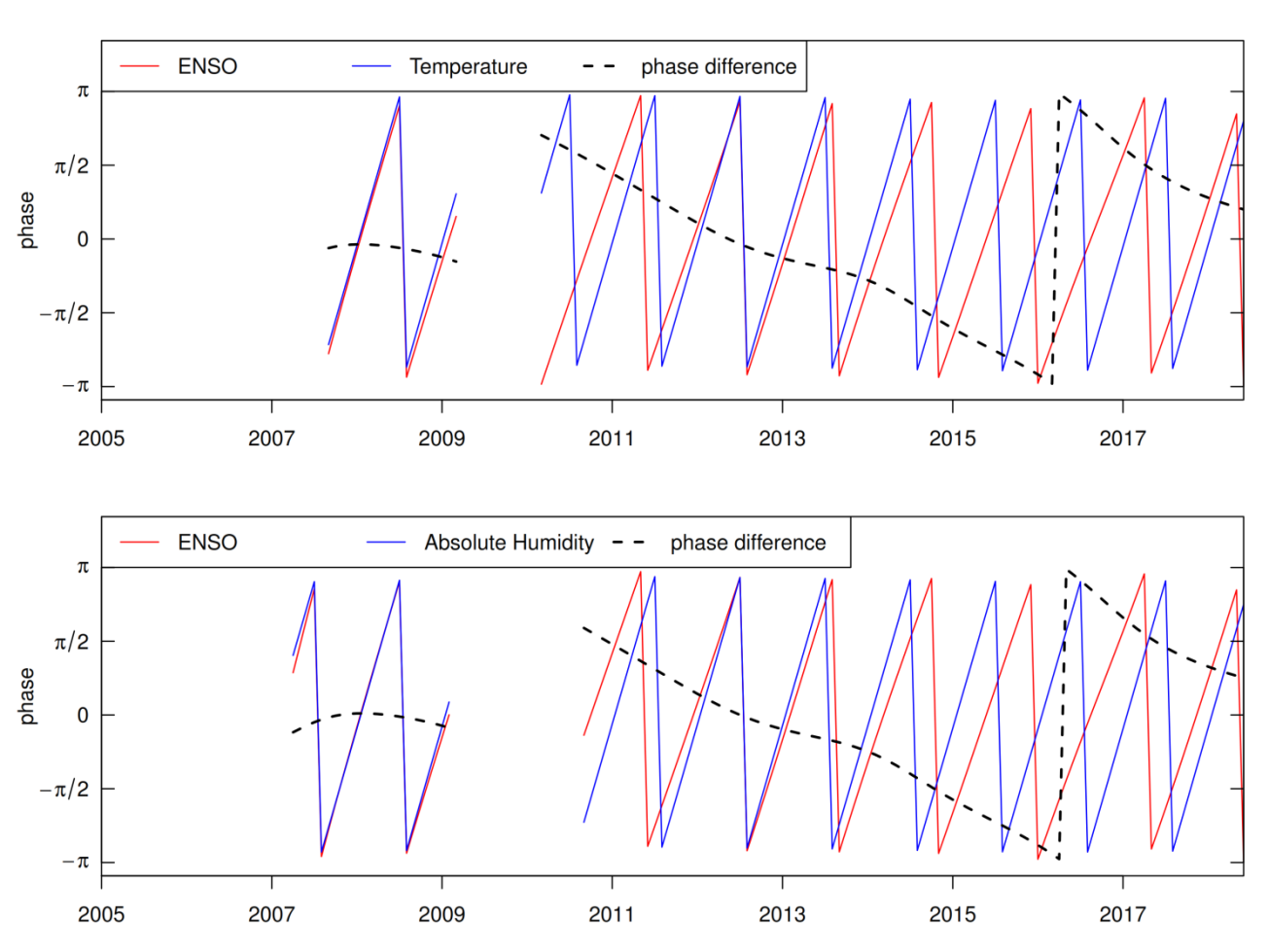
**

**Figure S5** The phase difference for ENSO-Temperature and ENSO-Absolute humidity at a periodicity of 1-years in New York State (one π represents 6 months)

**
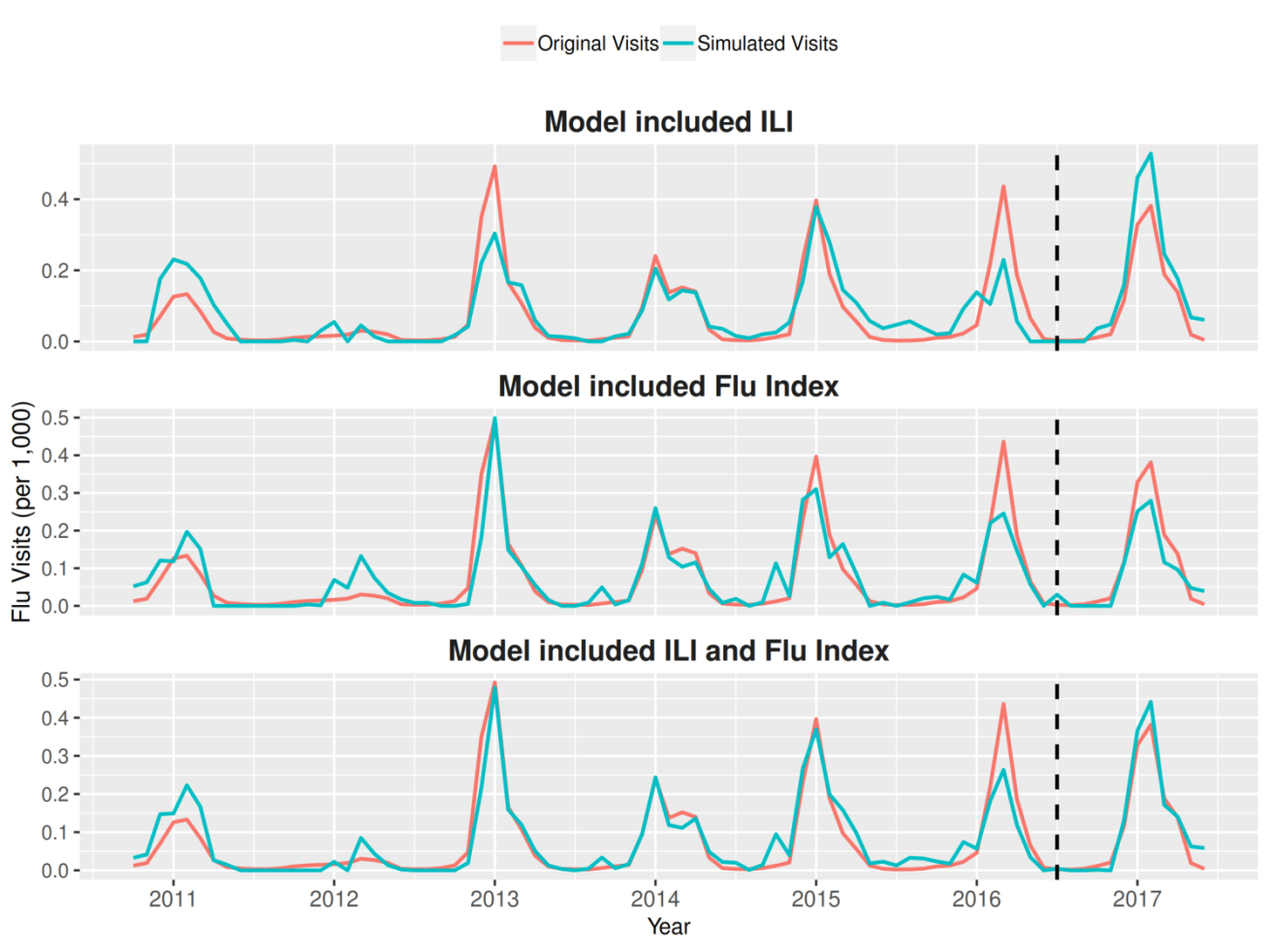
**

**Figure S6** The recorded influenza visits and simulated influenza visit in New York State from 10/2010 – 06/2017, based on three simulation models (fitting period: 10/2010 – 06/2016; simulation period: 07/2016 – 06/2017)

**
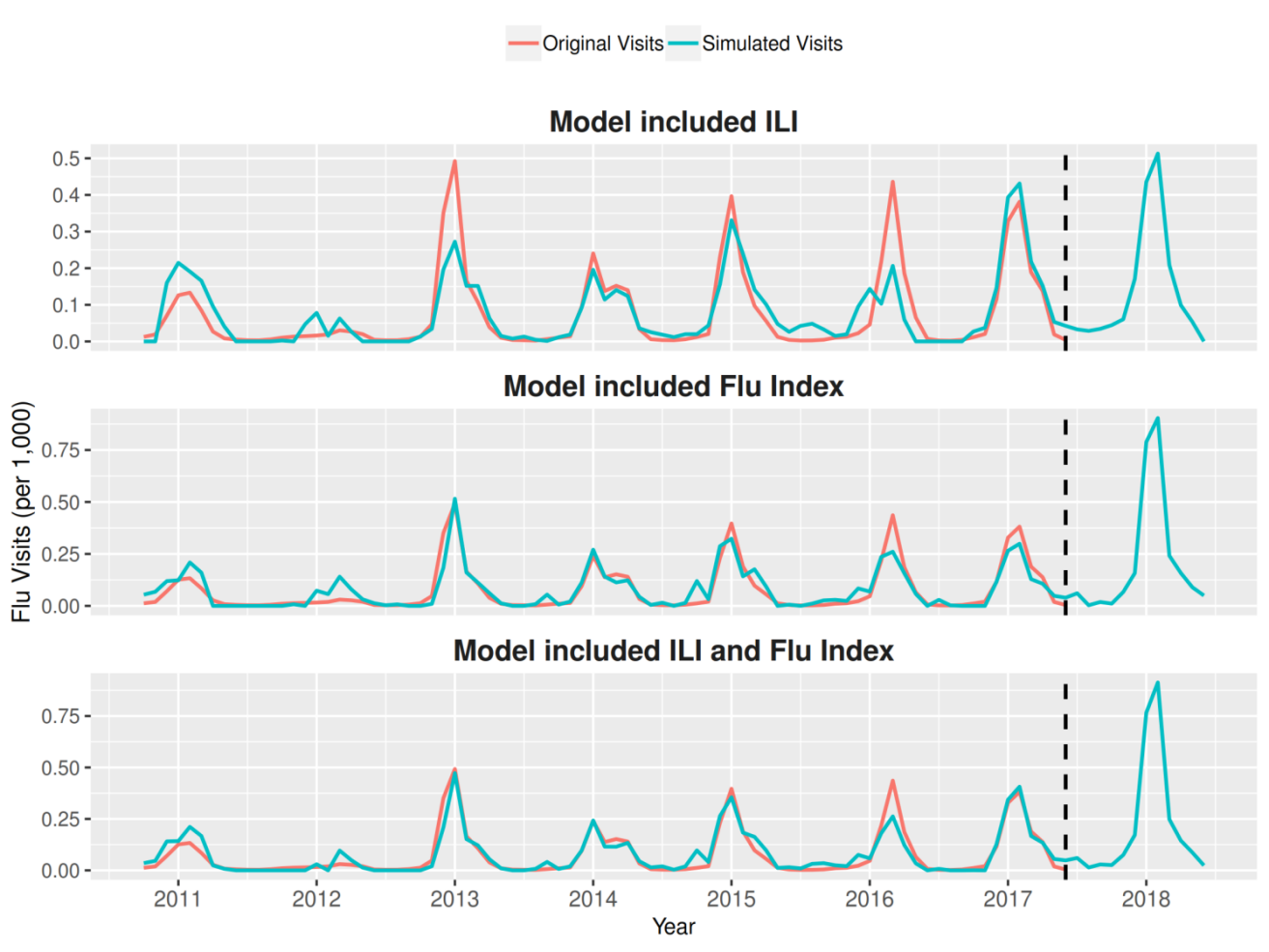
**

**Figure S7** The recorded influenza visits and simulated influenza visit in New York State from 10/2010 – 06/2018, based on three simulation models (fitting period: 10/2010 – 06/2017; simulation period: 07/2017 – 06/2018)

**Table S1.** Fitting accuracy for fitting and simulation for influenza incidence in New York State based on three Bayesian structural time series models

| Models | RMSE for fitting period of 10/2010 **–** 06/2016 | RMSE for simulation period of 07/2016 **–** 06/2017 | RMSE for fitting period of 10/2010 **–** 06/2017 |
| --- | --- | --- | --- |
| Model included Influenza-like illness | 0.057 | 0.066 | 0.056 |
| Model included Google flu index | 0.045 | 0.047 | 0.045 |
| Model included Influenza-like illness and Google flu index | 0.039 | 0.031 | 0.038 |
